# Myo-Inositol Limits Kainic Acid-Induced Epileptogenesis in Rats

**DOI:** 10.3390/ijms23031198

**Published:** 2022-01-21

**Authors:** Manana Kandashvili, Georgi Gamkrelidze, Lia Tsverava, Tamar Lordkipanidze, Eka Lepsveridze, Vincenzo Lagani, Maia Burjanadze, Manana Dashniani, Merab Kokaia, Revaz Solomonia

**Affiliations:** 1 Institute of Chemical Biology, School of Natural Sciences and Medicine, Ilia State University, 3/5 K. Cholokashvili Avenue, Tbilisi 0162, Georgia; manana.kandashvili.1@iliauni.edu.ge (M.K.); giga_gamkrelidze@iliauni.edu.ge (G.G.); lia.tsverava.2@iliauni.edu.ge (L.T.); tamar_lortkipanidze@iliauni.edu.ge (T.L.); eka_lepsveridze@iliauni.edu.ge (E.L.); vincenzo.lagani@iliauni.edu.ge (V.L.); 2 I. Beritashvili Center of Experimental Biomedicine, 14 L. Gotua Street, Tbilisi 0160, Georgia; burj_m@yahoo.com (M.B.); m.dashniani@yahoo.com (M.D.); 3 Biological and Environmental Sciences and Engineering Division, King Abdullah University of Science and Technology, Thuwal 23955, Saudi Arabia; 4 Epilepsy Centre, Department of Clinical Sciences, Lund University Hospital, SE-221 00 Lund, Sweden

**Keywords:** myo-inositol, kainic acid, epilepsy, epileptogenesis, electrographic seizures, learning and memory, glial fibrillary acidic protein, volume regulated anionic channel, protein tyrosine phosphatase receptor type R

## Abstract

Epilepsy is a severe neurological disease characterized by spontaneous recurrent seizures (SRS). A complex pathophysiological process referred to as epileptogenesis transforms a normal brain into an epileptic one. Prevention of epileptogenesis is a subject of intensive research. Currently, there are no clinically approved drugs that can act as preventive medication. Our previous studies have revealed highly promising antiepileptogenic properties of a compound–myo-inositol (MI) and the present research broadens previous results and demonstrates the long-term disease-modifying effect of this drug, as well as the amelioration of cognitive comorbidities. For the first time, we show that long-term treatment with MI: (i) decreases the frequency and duration of electrographic SRS in the hippocampus; (ii) has an ameliorating effect on spatial learning and memory deficit associated with epileptogenesis, and (iii) attenuates cell loss in the hippocampus. MI treatment also alters the expression of the glial fibrillary acidic protein, LRRC8A subunit of volume-regulated anion channels, and protein tyrosine phosphatase receptor type R, all expected to counteract the epileptogenesis. All these effects are still present even 4 weeks after MI treatment ceased. This suggests that MI may exert multiple actions on various epileptogenesis-associated changes in the brain and, therefore, could be considered as a candidate target for prevention of epileptogenesis.

## 1. Introduction

Epileptogenesis is a dynamic and multifactorial process of molecular, cellular, and network changes that cause certain structural and functional reorganization of the brain, in many cases induced by the precipitating events or insults. This process leads to the development of epilepsy—a disease that is characterized by spontaneous recurrent seizures (SRS) [1]. The International League against epilepsy defines epilepsy by the existence of two unprovoked seizures >24 h apart [2]. Approximately 1% of the world population suffers from epilepsy. Currently available antiseizure medications (ASMs) do not prevent or cure epilepsy and offer only symptomatic relief by suppressing SRS. However, up to 30% of the patients are refractory to ASMs [1,3,4,5].

It has been proposed that treatment strategies that could interfere with epileptogenesis would provide significant benefit by preventing or modifying the disease. Unfortunately, at present, there are no medications available that could effectively prevent the process of epileptogenesis or modify the disease in humans or experimental animals [1,3,4,5,6]. Even if antiepileptogenesis treatment would not fully prevent the development of the disease, but only modify it by decreasing SRS frequency and/or severity, it would still be considered as a significant achievement [4].

In our early studies, we have shown that water extract of *Aquilegia vulgaris* (plant widely used in Chinese and Tibetan folk medicine as antiepileptic and soporific medication) contains compounds affecting γ-aminobutyric acid A (GABA-A) receptors in vitro [7]. These compounds were identified as myo-inositol (MI) and sleep-inducing lipid oleamide. All following experiments were carried out with commercially available high purity MI and oleamide. MI completely prevents 3H-muscimol (a GABA-A receptor agonist) binding in rat brain-derived cell membranes, while oleamide increases 3H-Flunitrezepam (a specific ligand for the GABA-A receptor benzodiazepine site) binding approximately two-fold [7], suggesting that MI may be a GABA-A receptor agonist, while oleamide could represent a positive modulator of GABA-A receptors. Both of these actions would have seizure suppressant effects in the brain, since the number of antiseizure drugs have been shown to act as agonists of GABA-A receptors and enhance inhibition of neurons in the central nervous system [8]. The MI and its derivatives are also present in the CNS cells and are playing an important role in their function [9].

In line with this notion, in our previous studies, we have demonstrated that MI pretreatment significantly decreased severity of acute seizures induced either by pentylentetrazolium (PTZ) or by kainic acid (KA) in experimental animals [10], and prevents cell loss in the hippocampus after KA induced SE [11].We also found that MI has time- and concentration-dependent inhibitory effect on the evoked epileptiform after-discharges induced by local electrical stimulation, and hypothesized that MI, in addition to other known functions [9], may represent an endogenous anti-seizure agent in the CNS [12,13].

In the follow-up studies, we demonstrated that MI treatment attenuated molecular changes related to the KA-induced epileptogenesis [14,15]. Namely, KA-induced epileptogenesis was associated with a strong decrease (>60%) in the levels of AMPA-glutamate receptor GLUR1 subunit, calcium-calmodulin dependent protein kinase II (CaMKII), and gamma-2 subunit of GABA-A receptor in the hippocampus. All these changes were nearly completely prevented by MI daily treatment [14,15].

We also demonstrated that, in KA-induced post-SE epilepsy model in rats, MI reduces frequency and duration of behavioural (motor) SRS during and even 4 weeks after the treatment [16]. In addition, MI had normalizing effect on mi-RNA expression spectrum, mRNA levels of sodium-MI transporter and LRRC8A subunit of the volume regulated anionic channel [16]. This study indicated that the MI could potentially modify epileptogenesis.

To capitalize on these findings, in the present study, we investigated the effect of MI on electrophysiological, morphological, behavioural and biochemical changes tightly associated with epileptogenesis. We demonstrate that 4 week MI treatment right after KA-induced SE: (i) decreases the frequency and duration of electrographic (EEG) SRS in the hippocampus; (ii) improves spatial learning and memory deficit associated with epileptogenesis, even 4 weeks after MI treatment termination; (iii) MI attenuates cell loss in the hippocampus and (iv) alters the expression of the glial fibrillary acidic protein, LRRC8A subunit of volume-regulated anion channels (VRAC), protein tyrosine phosphatase receptor type R (PTPRR) in a manner that is expected to counteract the epileptogenesis process.

## 2. Results

### 2.1. Behavioural Analysis

All the experimental animals from both groups (KA + Saline [SAL] and KA + MI) exhibited spontaneous recurrent seizures (SRS). The mean number of SRS and mean duration of SRS per animal during 8-week monitoring period was higher in KA + SAL than KA + MI group (*p* = 0.005 and *p* = 0.002 respectively, see Supplementary Table S1).

### 2.2. Electrographic Seizures

#### 2.2.1. Number of Electrographic SRS

**Figure 1:**
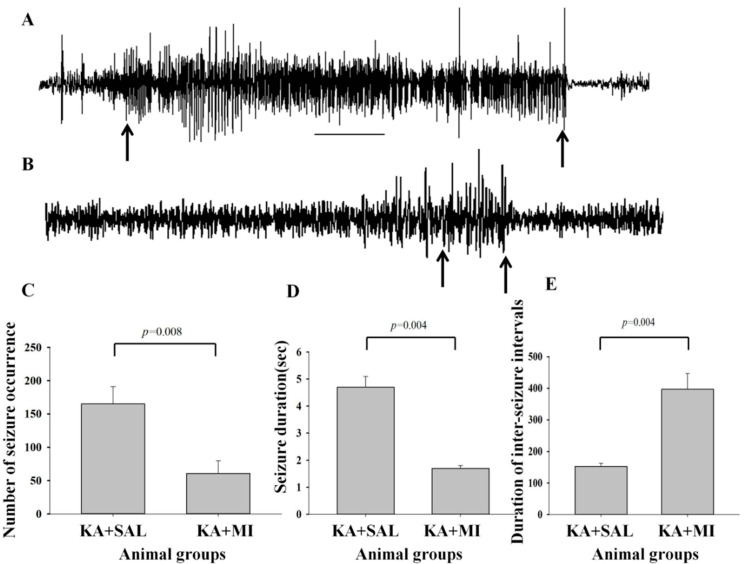
Electrographic seizures in the hippocampus of KA + SAL and KA + MI groups of rats. (**A**,**B**)—typical episodes of electrographic SRS in animals from KA + SAL and KA + MI groups respectively. Black arrows indicate the beginning and the end of the electrographic seizure episodes (**C**) mean number of electrographic SRS; (**D**) mean duration of electrographic SRS and (**E**) mean interval between electrographic SRS. Horizontal bar in A corresponds to 5 s. (**C**–**E**) On the abscissa is shown group type while on ordinate the characteristic of the electrographic SRS events. The error bars indicate standard error of the mean. (**C**) KA + MI group displayed significantly lower number of the electrographic SRS activity than KA + SAL group (**D**)—KA + MI group showed significantly shorter duration of the electrographic SRS than KA group. (**E**)—on the ordinate the average duration (sec) of the interval between the SRS events is shown. KA + MI group showed significantly longer intervals between the electrical seizure activity than KA + SAL group. Details of statistical analysis are provided in the manuscript.

The total number of electrographic SRS for each animal during monitoring sessions is reported in Supplementary Table S2. The typical SRSs recorded during the monitoring sessions is presented in Figure 1A,B. The mean total number of the electrographic SRS recorded during the monitoring sessions was 165.0 ± 25.7 in KA + SAL group, while in KA + MI was 60.9 ± 19.3 (Figure 1C).

#### 2.2.2. Duration of Electrographic SRS

The duration of the electrographic SRS is quantified with integer values and follows a non-normal distribution (Shapiro-Wilk normality test *p*-value < 2.2 × 10^−16^). The average duration of electrographic SRS in KA + SAL group was 4.7 ± 0.4 s and in KA + MI 1.7 ± 0.1 s. The generalized mixed model shows that animals in the KA + SAL group experienced significantly longer SRS events (*p*-value: of 0.004) (Figure 1D).

#### 2.2.3. Intervals between Seizure Electrical Activities

The length of the intervals between SRS events is also quantified with integer values and follows a non-normal distribution (Shapiro–Wilk normality test *p*-value < 2.2 × 10^−16^). The average value for the interval between electrical seizure activities for KA + SAL group was 153.0 ± 9.7 s. and for KA + MI was 397 ± 50.3 s, (Figure 1E). We again modeled the data using a Poisson generalized linear mixed regression, with group as fixed effect and animals as random effect. The model provides a significant *p*-value of 0.004 for the difference between the groups, with animals in the KA + MI group experiencing much longer intervals between SRS.

#### 2.2.4. Interictal Events

The interictal events distribution was also non-normally distributed (Shapiro-Wilk normality test *p*-value = 2.157 × 10^−6^). However, no significant difference between the two groups was detected by using a generalized mixed model approach (mean numbers of interictal activities ±sem; KA + SAL group 936 ± 300, KA + MI group 963 ± 154, *p* = 0.267).

### 2.3. Spatial Task Learning

#### Learning Escape Latency Time

It is well known that KA administration in rats impairs performance in the Morris water maze (MWM) learning and memory tasks [17,18]. We asked whether MI treatment could prevent this impairment, and indeed have shown that KA + MI group were performing significantly better in MWM. For the learning escape latency time two-way ANOVA analysis revealed that the effects of both factors (treatment and day) are highly significant, F(2,18) = 7.07, *p* = 0.0054 and F(3,60) = 31.11, *p* = 2.9 × 10^−12^, respectively. When measurements were gathered across the 4 days, Tukey’s *p*-values show no statistically significant differences between the CON + SAL and the KA + MI groups (T = 2.43, *p* = 0.062, DF = 18), or between the KA + SAL and the KA + MI groups (T = 1.258, *p* = 0.43, DF = 18), while the escape latency time of the CON + SAL group is significantly lower than the one of the KA + SAL group (T = 3.697, *p* = 0.0045, DF = 18).

**Figure 2:**
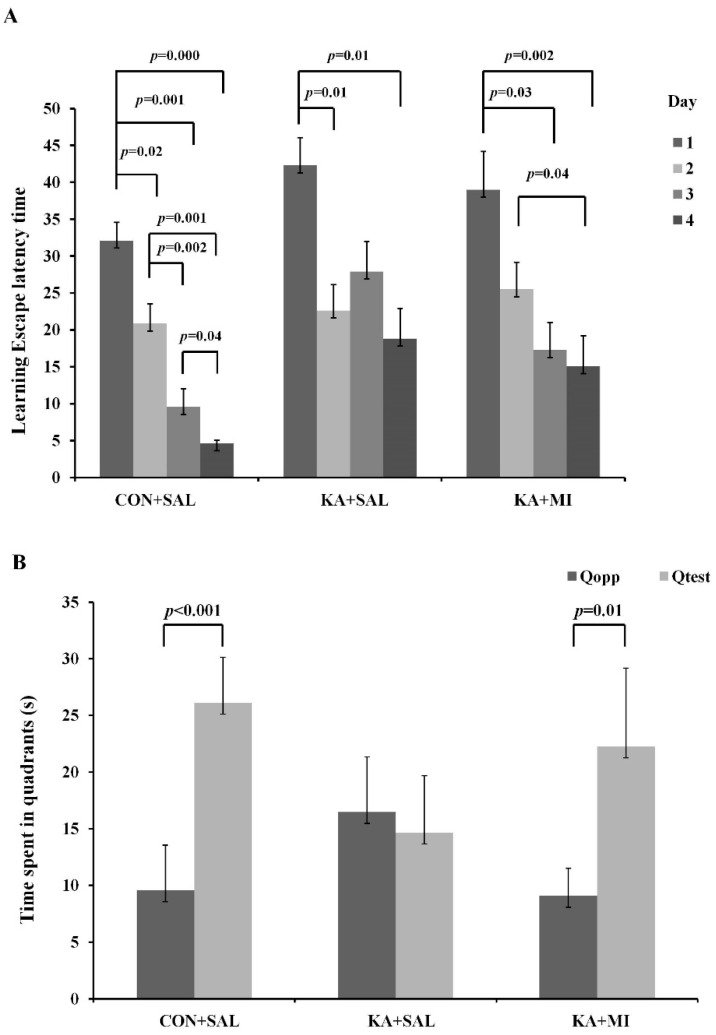
Spatial task learning and memory retention in MWM. (**A**)—distribution of the escape latency time (seconds ordinate) separated in treatment group and day. In each boxplot the central bar represents the median of the distribution, while the box covers the 0.25–0.75 interquartile range (IQR). Whiskers extend in both directions from the extremes of the box up to 1.5 times the IQR. (**B**)—Distribution of the time spent in each quadrant (seconds, *y*-axis) separated per treatment group. Distributions are represented through boxplots, as conducted in panel (**A**)”.

Time-dependent comparisons within each treatment group revealed that in the control group, each day is followed by a significant reduction in escape latency time with respect to the previous day (see Supplementary Table S3 and Figure 2). In the KA + SAL group, a significant reduction was observed on day 2 and 4 with respect to day 1, whilst on day 3 and 4 no improvements are detected as compared to the previous day (see Supplementary Table S3). For the KA + MI group a significant difference was observed between day 1 and days 3 and 4, as well as between day 2 and day 4 (see Supplementary Table S3). The velocity of swimming was not different between the groups. Thus, the dynamics of learning and learning efficiencies was different between KA + SAL and KA + MI groups, with better capacity in MI treated group.

### 2.4. Spatial Memory

#### Time Spent in Quadrant I and IV

MI treatment also have a positive effect on the MWM memory task. The ANOVA analysis identified both the quadrant and the interaction between treatment and quadrant as significant, F(2,36) = 39.15, *p* = 3.1 × 10^−7^ and F(2,36) = 14.4, *p* = 2.5 × 10^−5^, respectively. Tukey’s corrected *p*-values showed no statistically significant differences for the time spent in quadrant I for the three groups, while control animals spend significantly more time in quadrant IV than the KA + SAL group (*p* = 0.001).

Both the CON + SAL and KA + MI groups show significant difference for the time spent in the two quadrants (*p* < 0.001 and *p* = 0.001, respectively, Figure 2B). No difference is detected in the KA + SAL group indicating that no spatial memory has been formed in this group after training sessions.

Finally, the adjusted one-tailed *t*-test and *p*-values indicate that both the control and the KA + MI groups remain in quadrant IV for more than 15 s (*p* < 0.001 and *p* = 0.048, respectively).

### 2.5. Cell Counts

In our previous study, we showed neuronal cell loss observed 24 h after KA induced SE in the hippocampus, and MI treatment had a protective effect on cell death [19]. Here we have analyzed whether KA and MI treatment effects in the hippocampus was still observed 8 weeks after SE. This analysis revealed that in all three hippocampal subfields, there was a significant reduction in number of neurons in KA + SAL group, which was partially prevented by MI treatment at this timepoint as well. This effect was more pronounced in cornu ammonis 1 (CA1) and cornu ammonis 3 (CA3) subfields (Two-way ANOVA with factors: 1. treatment conditions and 2. hippocampal subfields demonstrated a significant effect for both: F(2,53) = 60.76, *p* < 0.0001 and F(2.53) = 2372.51, *p* < 0.0001 respectively).

#### 2.5.1. CA1

**Figure 3:**
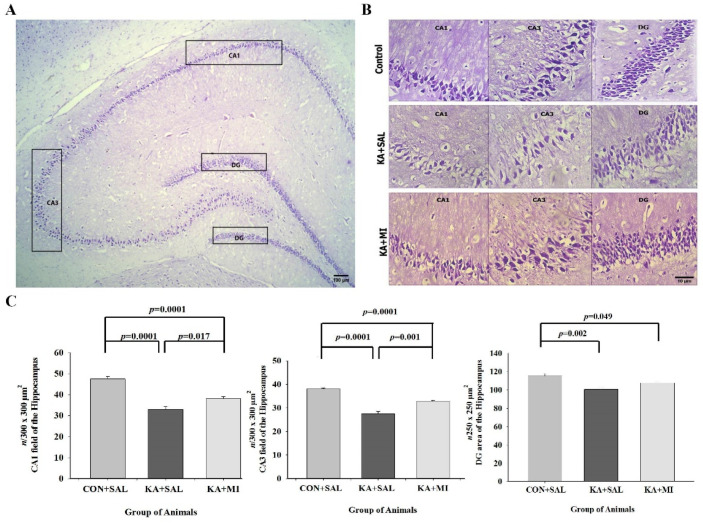
MI treatment prevents the KA-induced cell decrease in the hippocampus-the morphological changes in hippocampus subfields of three experimental groups. (**A**)—the view of hippocampus subfields, where cell counts were performed; (**B**)—representative photomicrographs of Nissl staining sections from the hippocampus of Control, KA + SAL and KA + MI group of rats; (**C**) Mean numbers of neurons in the CA1, CA3 and DG subfields of hippocampus in three different group of animals.

KA treatment induced strong and significant reduction of neuronal cell numbers as compared to CON + SAL and KA + MI groups (Figure 3B,C). The number of neurons was also decreased in KA + MI group as compared to CON + SAL group. Thus, MI treatment partially rescued the KA induced cell loss in CA1 area of the hippocampus. [One way ANOVA treatment factor F(2,17) = 38.04, *p* < 0.0001. CON + SAL vs. KA + SAL group (~30% T = 8.00, *p* = 0.0001, DF = 10). CON + SAL vs. KA + MI group (~20%, T = 6.49, *p* = 0.0001, DF = 10). KA + MI vs. KA + SAL group (T = 2.87, *p* = 0.017, DF = 10)].

#### 2.5.2. CA3

The effects of MI treatment on neuronal cell numbers in CA3 was similar to that in the CA1. The strong reduction of neuron numbers in KA + SAL group was significantly rescued by MI treatment, Figure 3B,C. [One-way ANOVA factor of treatment-F(2,17) = 53.13, *p* = 0.0001. KA + SAL vs. CON + SAL; T = 9.09, *p* < 0.0001 and KA + SAL vs. KA + MI groups; T = 4.39, *p* = 0.001, DF = 10 for both comparisons]. The still reduced number of neurons as compared to CON + SAL group was also observed in CA3 of KA + MI group (T = 9.02, *p* < 0.0001, DF = 10).

#### 2.5.3. Dentate Gyrus (DG)

This hippocampal subfield in KA + SAL group was also characterized by the reduced number of neurons, demonstrating the rank order of the means was similar to CA1 and CA3 subfields: CON + SAL > KA + MI > KA + SAL (One-way ANOVA factor of treatment F(2,17) = 9.45, *p* = 0.002). The significant decrease in number of neurons was observed in KA + SAL as compared to CON + SAL group (T = 4.20, *p* = 0.002, Figure 3C). The difference between CON + SAL and KA + MI was only marginally significant (T = 2.23, *p* = 0.049, DF = 10), whereas the difference between the KA + MI and KA + SAL groups was not significant on 2-tailed test (T = 2.19, *p* = 0.053, DF = 10).

The photomicrographs demonstrate the obvious decrease in neuronal cells in CA1, CA3 and DG subfields of hippocampus

As a result of KA + SAL treatment and partial rescue of the cells in KA + MI group. C Mean number of neurons (number of cell counts per counting frame area (250 × 250 μm^2^)) in the CA1, CA3 and DG subfields of the hippocampus. Error bars represent the standard errors of the means. Scale bar = 15. The details of statistical analysis are provided in the manuscript.

### 2.6. Biochemical Changes

#### 2.6.1. GFAP

Glial fibrillary acidic protein (GFAP) is an intermediate filament protein that is primarily expressed in astrocytes [20] and is strongly upregulated after CNS injuries. Reactive astrogliosis has been associated with epilepsy [21]. We have studied GFAP protein levels 8 weeks after KA induced SE and MI treatment.

##### Hippocampus

**Figure 4:**
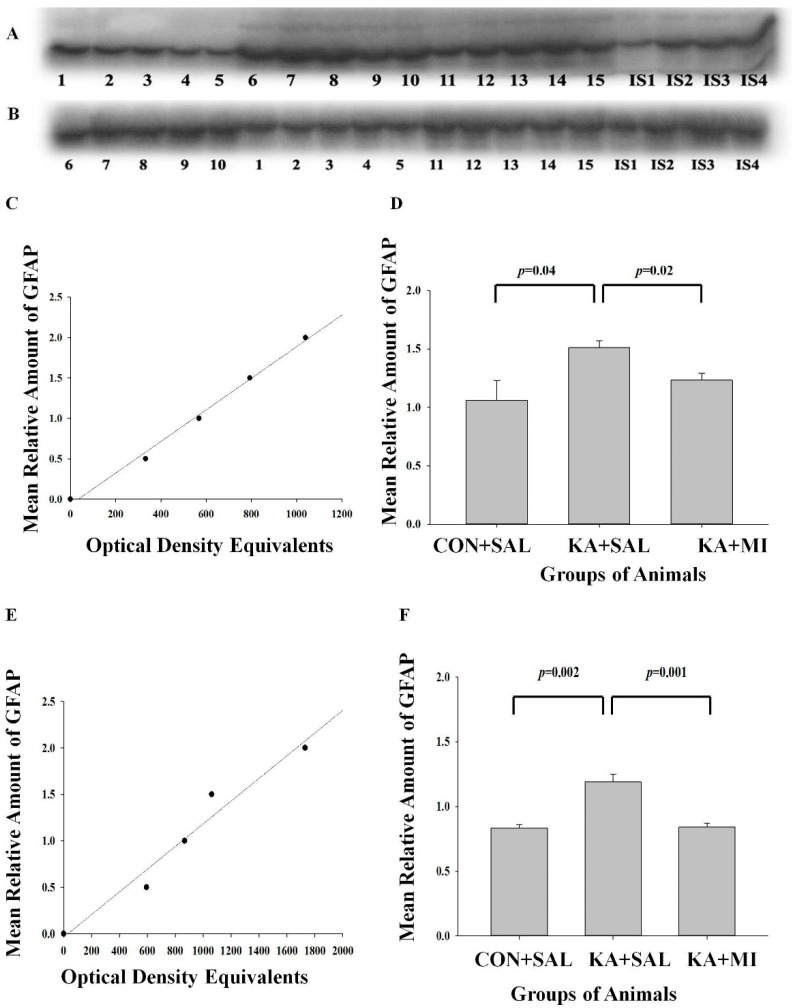
MI treatment reduces KA-induced upregulation of GFAP in the hippocampus and neocortex. (**A**,**C**,**D**) Hippocampus, (**B**,**E**,**F**) Neocortex. (**A**,**B**) Sample films; each lane corresponds to one sample. Lanes 1–5 are from CON + SAL group; lanes 6–10 from KA + SAL group and lanes 11–15 from KA + MI group. Lanes IS-1-IS4 internal standards containing, respectively, 15, 30, 45, and 60 μg protein. (**C**,**E**) Calibration plot (lines fitted by linear least-squares regression) and (**D**,**F**) Mean levels (mean ± standard error of the mean) of GFAP in hippocampus and neocortex respectively. The details of statistical comparisons are provided in manuscript.

Highest levels of GFAP as measured by WB was observed in KA + SAL group, which significantly exceeded CON + SAL as well as KA + MI groups, whereas CON + SAL and KA + MI were not different from each other (Figure 4A,C). [One way ANOVA treatment factor F(2,14) = 4.02, *p* = 0.046. KA + SAL vs. CON + SAL group T = 2.45, *p* = 0.04, DF = 8; KA + SAL vs. KA + MI group T = 2.89, *p* = 0.02, DF = 8].

##### Neocortex

The differences between the groups followed closely the pattern observed in the hippocampus. The amount of GFAP was significantly higher in KA + SAL group as compared to the CON + SAL and KA + MI groups (Figure 4D,E), while The CON + SAL and KA + MI groups were not different from each other [One-way ANOVA, factor of treatment F(2,14) = 19.96, *p* = 0.0001. KA + SAL vs. CON + SAL group T = 4.70, *p* = 0.002, DF = 8; KA + SAL vs. KA + MI group T = 5.18, *p* = 0.001. For both comparison DF = 8].

Thus, in both brain regions KA induced increase in GFAP levels, whereas MI treatment had a long-lasting inhibitory effect on the increase gliogenesis.

#### 2.6.2. LRRC8A Subunit of VRAC

We have previously shown that in the hippocampus, the LRRC8 mRNA is increased in KA + SAL group as compared to KA + MI and CON + SAL groups [16]. Here, we asked whether these changes in mRNA were also translated in the LRRC8 protein levels since this may not follow the mRNA changes in a linear way [22].

##### Hippocampus

**Figure 5:**
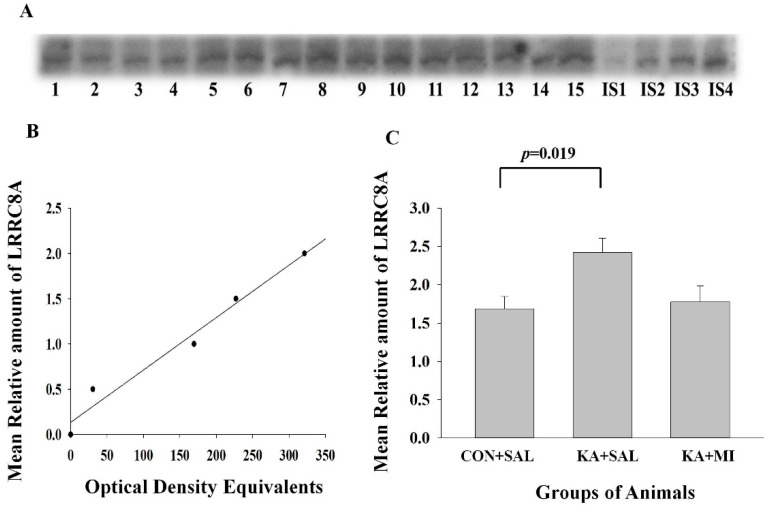
LRRC8A protein is increased in the hippocampus of KA + SAL group. (**A**) Sample film, (**B**) Calibration plot (lines fitted by linear least-squares regression) and (**C**) Mean levels (mean ± sem). Each lane corresponds to one sample. Lanes 1–5 are from CON + SAL group; lanes 6–10 from KA + SAL group and lanes 11–15 from KA + MI group. Lanes IS-1-IS4 internal standards containing, respectively, 15, 30, 45, and 60 μg protein. The details of statistical analysis are provided in the manuscript.

The mean levels of LRRC8 measured by WB was highest in KA + MI group and significantly exceeded that in the CON + SAL group. There was no significant difference between KA + MI and CON + SAL groups [One-way ANOVA treatment factor F(2,14) = 4.42, *p* = 0.037, KA + SAL vs. CON + SAL group T = 2.92, *p* = 0.019, Figure 5]. Thus, KA-induced epileptic state is associated with upregulation of LRRC8 protein levels which corresponds to previously shown increased mRNA levels [16], whilst MI treatment attenuates this increase.

##### Neocortex

No differences between the groups in the levels of LRRC8 were observed [CON + SAL 1.55 ± 0.1, KA + SAL 1.39 ± 0.09 and KA + MI 1.42 ± 0.11].

#### 2.6.3. Protein Tyrosine Phosphatase Receptor Type R (PTPRR)

##### Hippocampus

Our previous data show that the levels of the mi-RNA-6216 were significantly decreased in the hippocampus of KA + SAL group [14]. Protein tyrosine phosphatase receptor type R (PTPRR) mRNA is one of the targets of the miRNA-6216 (see http://www.mirdb.org/, accessed in 1 May 2019). This enzyme is the negative regulator of the extracellular regulated kinases, which when activated may cause epilepsy and seizures [23,24,25,26,27].

**Figure 6:**
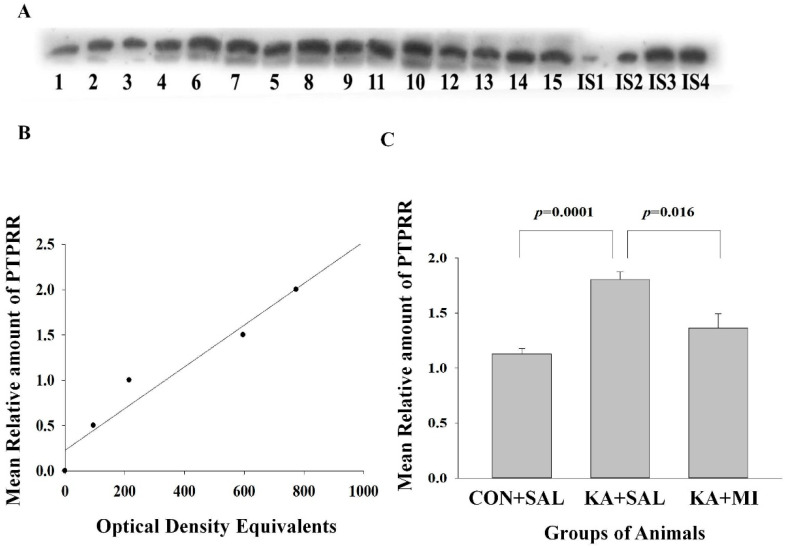
PTPRR protein is increased in the hippocampus of KA + SAL animals. (**A**) Sample film, where each lane corresponds to one sample. Lanes 1–5 are from CON + SAL group; lanes 6–10 from KA + SAL group and lanes 11–15 from KA + MI group. Lanes IS-1-IS4 are internal standards containing, respectively, 15, 30, 45, and 60 μg protein. (**B**) Calibration plot (lines fitted by linear least-squares regression); (**C**) Mean levels of PTPRR (mean ± sem).

The mean levels of PTPRR as measured by WB were the highest in the KA + SAL group as compared to CON + SAL and KA + MI groups. No significant differences were detected between CON + SAL and KA + MI groups (Figure 6C). [One-way ANOVA, treatment factor F(2,14) = 14.73, *p* = 0.001. KA + SAL vs. CON + SAL group T = 7.67, *p* = 0.000 and KA + SAL vs. KA + MI group T = 3.03, *p* = 0.016 for both comparisons DF = 8]. Thus the PTPRR protein changes are reciprocal to miR-6216 changes in the hippocampus [16], which may indicate that miR-6216 could be implicated in the processes of KA-induced epileptogenesis, at least by targeting PTPRR mRNA translation. MI treatment prevents the decrease in miRNA-6216 levels and, correspondingly, counteracts the increase in PTPRR.

##### Neocortex

One-way ANOVA did not reveal any statistically significant changes in the levels of PTPRR.

#### 2.6.4. Doublecortin

KA-induced SE is associated with the increased levels of doublecortin, one of the markers of neurogenesis [28].

**Figure 7:**
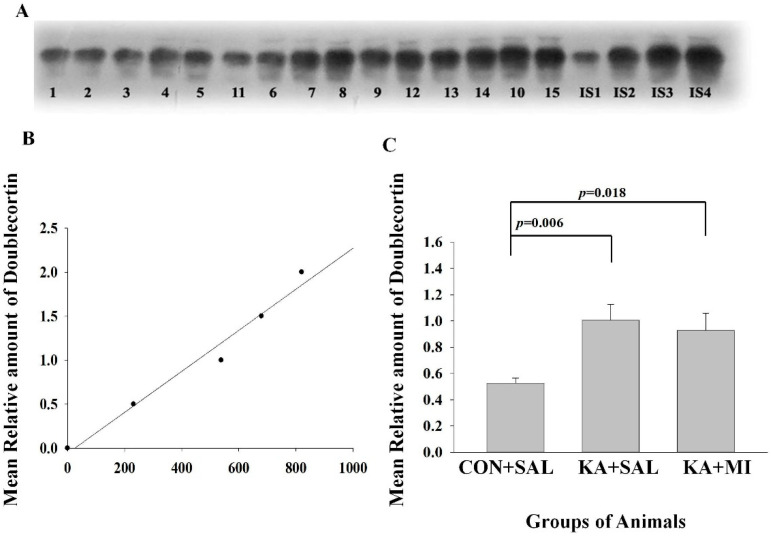
Hippocampus, Doublecortin levels are increased both in KA + SAL and KA + MI groups. (**A**) Sample film, where each lane corresponds to one sample. Lanes 1–5 are from CON + SAL group; lanes 6–10 from KA + SAL group and lanes 11–15 from KA + MI group. Lanes IS-1-IS4 internal standards containing, respectively, 15, 30, 45, and 60 μg protein. (**B**) Calibration plot (lines fitted by linear least-squares regression); (**C**) Mean levels of Doublecortin (mean ± sem) The details of statistical analysis are provided in the manuscript.

The mean levels of Doublecortin were significantly increased in KA + SAL and KA + MI groups as compared to CON + SAL group. There was no statistically significant difference between the KA-treated groups [One-way ANOVA (F2,14) = 5.92, *p* = 0.016; KA + SAL vs. CON + SAL, T = 3.72, *p* = 0.006 and KA + MI vs. CON + SAL group T = 2.96, *p* = 0.018, for both comparisons DF = 8, Figure 7C]. No Significant differences were detected between the groups in the neocortex.

## 3. Discussion

The present data demonstrate for the first time that MI has a long-term effect on several features associated with epileptogenesis and ictogenesis: (i) electrophysiological characteristics; (ii) learning and memory process; (iii) morphological changes, and (iv) several molecular changes are discussed below in detail. It is remarkable that all these effects are maintained over a long period of time after termination of MI treatment. This led us to suggest that MI counteracts epileptogenesis, leading to disease modification. These findings are in line with our previous report showing the long-term effects of MI on behavioral SRS, as well as alterations in some of the biochemical processes associated with epileptogenesis after KA-induced SE [16].

### 3.1. Electrophysiology

In animal models of temporal lobe epilepsy (TLE), epileptogenesis is associated with the appearance of specific pathological electrographic activity in limbic structures [29,30,31,32,33]. These include interictal spikes (IIS) and focal electrographic seizure-like events that are preceding generalized spontaneous recurrent seizures (SRS) observed also at the behavioral level [34]. Here, we have demonstrated that chronic exposure to MI right after KA-induced SE decreases the frequency and duration of the focal electrographic SRS at later stages of epileptogenesis, which is maintained even after the MI treatment has been terminated. This outcome is in agreement with our previous results demonstrating the protective effect of MI on molecular, cellular and behavioral changes associated with epileptogenesis [14,15,16]. The hippocampal formation, due to its extensive recurrent connections and intrinsically bursting pyramidal neurons, is known to generate focal seizure-like events [34]. This activity can intensify, spread and recruit the cortical and related thalamo-cortical circuits that have been shown to play a critical role in generalized seizures [34,35]. Our results suggest that MI can act by suppressing focal electrographic seizure-like events in the temporal lobe early during epileptogenesis, thus mitigating the spread and generalization of seizures manifested as muscle convulsions at the behavioral level. However, the direct inhibitory effect of MI on the generalized SRS, involving thalamo-cortical network cannot be ruled out.

### 3.2. Learning and Memory

It is well known that rats administered with KA exhibit poor performance in learning and memory tasks assessed by Morris water maze (MWM) [17,18]. In our study, animals after KA-induced SE exhibited poor cognitive performance in these tasks. However, when these animals were treated with MI, a beneficial effect on learning efficiency/kinetics and memory retention was observed. During training, the control group was significantly better at performing compared to epileptic animals, but those treated with MI were similar to the controls. It is well known that hippocampus plays critical role in the spatial learning and memory [36]. Thus, MI treatment counteracts this comorbidity, with long-lasting effects even after the treatment. This effect is most likely the result of the seizure suppression by MI.

### 3.3. Morphology

Our previous study has shown that 24 h after KA-induced SE neuronal cell loss occurs in the CA1 and CA3 subfields of hippocampus, which is prevented by MI treatment [19]. The results of the present study are in agreement with these observations and indicate that MI treatment has a long-lasting beneficial effect on the KA-induced hippocampal cell loss. We presume that amelioration of the cell loss preserves overall hippocampal structure and prevents development of the aberrant neuronal circuits due to axonal sprouting that may contribute to epileptogenesis and SRS.

### 3.4. Molecular Changes

#### 3.4.1. GFAP

In response to the central nervous system (CNS) insults, astrogliosis is initiated as an adaptive, beneficial process for tissue protection (reviewed in [37]. However, under specific circumstances it could induce harmful effects, such as exacerbation of inflammation or the inhibition of axon regeneration by astrocyte scars [37]. Reactive astrogliosis has been associated also with epilepsy [21]. Expression of GFAP is also increased after KA induced SE [38,39]. In the present study, the level of GFAP expression, a marker of astrogliosis, was elevated both in the hippocampus and neocortex of epileptic animals. MI treatment prevented this elevation in both structures, and the levels of GFAP were not significantly different form the control group. Thus, MI treatment has a long-lasting effect and nearly normalizes and prevents processes of astrogliosis as measured by GFAP expression. Since repeated seizures have been shown to increase GFAP expression (e.g., [40]), the reduced number of electrographic as well as behavioral seizures in MI-treated epileptic animals as compared to non-treated group could explain the normalized levels of GFAP. Thus, one could conclude that elevated pathological astrogliosis in epileptic animals is effectively prevented by MI treatment.

#### 3.4.2. LRRC8A Subunit of VRAC

In our previous study [16], we demonstrated, for the first time, that the mRNA coding for LRRC8A subunit of VRAC is upregulated in the hippocampus of epileptic animals. This upregulation was significantly decreased by MI treatment and the mean value of this mRNA in MI-treated group was not different from the values of the other two control groups [16]. However, as reviewed in [22] changes in mRNA expression may not always reflect the changes in protein levels. Increased mRNA levels represent a potential need in protein synthesis, and, therefore, should not be considered identical to actual final protein expression [22]. Thus, in the present study, we have demonstrated, importantly, that also protein levels of VRAC are elevated after KA-induced SE. VRAC plays a significant role in regulating the size of the cells by transporting chloride ions and various organic osmolytes across the plasma membrane [41]. In the CNS, VRAC is expressed both by neurons and astrocytes, and in both cell types, it is similarly activated by hypo-osmolar or excitotoxic conditions [42]. Epileptiform activity leads to the massive influx of Na^+^, Ca^2+^, and Cl^−^ ions into the cells resulting in associated inward flow of the water, cellular swelling, and extracellular space reduction [43,44,45]. Extracellular space reduction, in turn, enhances neuronal excitability [46,47]. As mentioned above, the number of electrographic as well as behavioral seizures are increased in epileptic animals, and it could be proposed that upregulation of LRRC8A protein, which is a subunit of VRAC, could be a compensatory reaction to counteract cell-swelling; thereby, limiting tissue damage and potentially also seizures generation/propagation.

#### 3.4.3. PTPRR

In our previous studies, comparative mi-RNA profiling of hippocampus in KA + SAL, KA + MI and CON + MI groups revealed more than 70 differentially expressed mi-RNAs across the groups [16]. For one of them, namely mi-RNA6216, further quantitative validation studies were conducted, demonstrating significant downregulation in the hippocampus of KA + SAL group as compared to CON + SAL or KA + MI groups [16]. As the mi-RNAs are inhibiting the process of mRNA translation, it was predictable that targets of mi-RNA6216 should be upregulated in the hippocampus of KA + SAL group. In the present study, we have chosen PTPRR from these targets, because by its tyrosine phosphatase activity is in upstream of extracellular regulated kinases (ERK–part of mitogen-activated protein kinase signaling cascade) and negatively regulates them [23]. Since ERK activation causes seizures and epilepsy [24,25,26], it seemed interesting to investigate PTPRR regulation during epileptogenesis. Indeed, the level of PTPRR protein was increased significantly in the hippocampus of KA + SAL group, suggesting that it might be a compensatory mechanism to counteract epileptogenesis and SRS. However, increased levels of PTPRR and resulting inhibition of ERK could have a negative impact on learning and memory since increased ERK activity is necessary for memory stabilization (reviewed by [48]. Therefore, the PTPRR upregulation may in fact contribute to impaired learning and memory in the epileptic animals.

#### 3.4.4. Doublecortin

It is well known that adult neurogenesis is altered dramatically during epileptogenesis, e.g., for review [49]. Some data points to the pro-epileptogenic role for adult-born neurons in the hippocampus. It was shown that aberrant adult neurogenesis is sufficient to induce spontaneous seizures in an otherwise intact animal [50]. Other data indicate that seizure-induced, non-aberrant neurogenesis may play a positive compensatory role in SE models [51]. Although Doublecortin is a marker of neurogenesis [28], in the present study, due to measuring total Doublecortin levels with WB, it is not possible to distinguish which type of neurogenesis is affected by SE. In any case, MI was not able to normalize levels of Doublecorin; therefore, it seems unlikely that MI effect is mediated by alterations in neurogenesis.

### 3.5. Other Possible Mechanisms of MI-Induced Modulation of Epileptogenesis

#### 3.5.1. Phosphoinositide Signaling Pathway

This signaling pathway is involved in the regulation of electrical activity of neurons via uptake of MI. Treatment of cultured sympathetic neurons by MI leads to its active transport into the cells, increased levels of Phosphatidylinositol 4,5-bisphosphate and enhanced muscarinic acetylcholine receptor modulated potassium current (M potassium conductance) [52]. MI-induced upregulation of the M potassium conductance decreases excitability of the sympathetic neurons [52]. M potassium conductance is present in the membrane of a variety of cells, including hippocampal pyramidal neurons.

It is noteworthy that M potassium channels colocalize with sodium-MI-transporter (SMIT) in the plasma membrane of the hippocampal pyramidal neurons [53]. We have previously shown that MI treatment leads to the upregulation of SMIT mRNA in epileptic animals, but the increase in MI treated animals is 5 times higher than in KA + SAL group [16]. Elevated SMIT levels should theoretically lead to the increased accumulation of MI intracellularly. It is tempting to hypothesize that a SMIT-mediated increase in MI concentration inside the neurons acts on the M channels and decreases excitability of the pyramidal neurons in a dose-dependent manner [13]. A long-term increase in the expression of SMIT mRNA during KA-induced epileptogenesis could therefore contribute to the disease-modifying effect of MI [16].

#### 3.5.2. MI Action on GABAA Receptors

In our previous experiments we have shown that MI completely displaces 3H-muscimol binding (GABA-A receptor agonist interacting with GABA binding site) from rat brain membranes in vitro [7]. Thus, it is possible that MI antipepileptogenic action could be mediated via potentiation of GABA-A receptor signaling. However, this remains to be tested in specific experimental conditions.

#### 3.5.3. Limitations of the Study and Future Research Directions

The limitation of the present study is the usage of only one dose of MI. This dose was estimated to be minimal for MI effects on convulsions [12]. In future studies, we plan to carry out comparative experiments with various increasing doses of MI. Such an approach will yield, automatically, the larger number of animals for 30 mg/kg dose of MI.

## 4. Materials and Methods

### 4.1. Animals

Animals were housed individually and maintained under 12 h light/12 h dark cycle and had a free access to the food and water. Experimental design was approved by the Bioethics committee of I.Beritashvili Centre of Experimental Biomedicine (Protocol N03/01.11.2019).

### 4.2. KA-Induced SE

Male Wistar rats, 2.5–3 months of age, received a single intraperitoneal (IP) injection of kainite (KA; 10 mg/kg, Abcam, Cambridge, UK, Cat.N. 120100) dissolved in saline [14,15,16,19,54]. After injection, each animal was placed into an individual plastic cage for observation for 4 h. Seizures were scored according to a modified Racine scale from 0 to 6: (0) no motor seizures; (1) freezing, staring, mouth, or facial movements; (2) head nodding or isolated twitches, rigid posture; (3) tail extension, unilateral–bilateral forelimb clonus; (4) rearing, in which the rat sits in an immobile state on its rear limbs with one or both forepaws extended; (5) clonic seizures, loss of posture, jumping, and forepaws extended; (6) tonic seizures with hind limb extension [55,56]. For further experiments, only animals that exhibited recurrent behavioral seizures scored as 4–6 and at least 60 min of total duration within 4 h observation period, were selected. This selection was applied to ensure induction of epileptogenesis and consequently development of spontaneous recurrent seizure (SRS), i.e., epilepsy [57].

**Figure 8:**
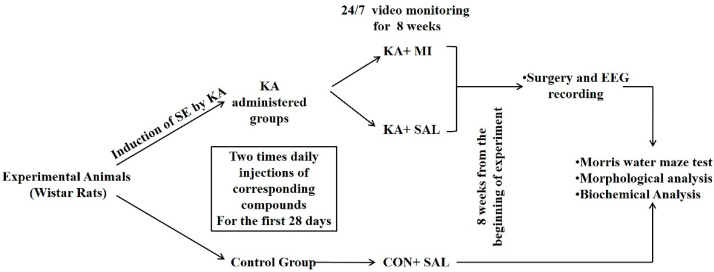
Schematic representation of the experimental design (see detailed description in the text).

In each series of experiments, half of the selected KA-treated rats were injected with MI (30 mg/kg, KA + MI group) and another half with saline (0.9% NaCl sterile solution, 1 mL/kg KA + SAL group). IP injections (twice per day) started 4 h following KA treatment and continued for 28 days from the beginning of experiment. The control group of animals (CON + SAL) received intraperitoneal injection of saline (0.9% NaCl solution, 1 mL/kg) twice daily for 28 days as KA treated group animals. Schematic representation of the experimental design is provided on Figure 8.

### 4.3. Video-Monitoring

Animals were housed individually and maintained under 12 h light/12 h dark cycle. Animal behavior was monitored 24/7 by infrared video cameras (IR_IP66_ HIK VISION, Hangzhou, China). Digital video files were recorded with Digital Video Recorder (HIK VISION, DS-7316HLS, Hangzhou, China) and kept on removable high-capacity hard disks. Recordings were reviewed offline (played at 4X speed) to detect any behavioral seizure activity according to a modified Racine scale described above [55,56]. Only seizures of grades 4–6 were identified and evaluated, since lower seizures grades can easily be confused with normal behavior.

### 4.4. Surgery and EEG Recording

At 28 days after termination of treatment with MI or saline, a steel bipolar electrode was implanted in the right dorsal hippocampus under ketamine anesthesia using rat brain atlas [58]. The following coordinates were selected for the placement of the electrode in the right hippocampus: 4.5 mm caudally from the bregma, 2.8 mm laterally from the mid-line, 3 mm ventral from the skull surface. The animals were allowed to recover for 4 days after surgery and the hippocampal EEG was recorded for 3 following days, for 3 h per day. Recording periods were alternated in the morning and afternoon between KA + SAL and KA + MI rats to ensure that each group was recorded for a defined period of the day for equal amount of time.

An EEG was recorded with an amplifier (Pinnacle Technology, Ottawa Lake, USA), low-pass filtered at 50 Hz and acquired with the software Sirenia (Pinnacle Technology, Ottawa Lake, USA). The duration, frequency and number of electrographic SRS were analyzed. The SRS activity was identified as high frequency of spike-and wave activity with the amplitude exceeding the background activity at least twice. Additionally, the number of interictal spikes was evaluated. Interictal spikes were identified as brief, sharp negative or negative positive deflections of potentials with duration less than 150 ms and the amplitude exceeding the background activity at least three times.

In total 7 rats were recorded from KA + SAL group and 7 rats from KA + MI group. These rats were randomly chosen from two series of independent experiments of KA treatment cohorts of animals.

The correct locations of implanted bipolar electrodes were verified by post hoc histological examination of the brain sections (see Supplementary Figure S1).

### 4.5. Morris Water Maze (MWM)

MWM test (Morris et al., 1982) was used to study spatial learning and memory of the KA + SAL, KA + MI and CONT + SAL rats. MWM test was conducted on the same 7 animals from each KA + SAL and KA + MI group, which were used in electrophysiological studies (see above).

Animal training/testing was carried out in an orientation-rich environment where the space around the Morris water tank included: pictures, figures, lighting. The diameter of the water tank was 1.5 m and the height—0.5 m. During the experiments, the pool was filled out with opaque (light gray) water with a temperature of 23 °C. The water tank was marked by four sectors corresponding to its location relative to the Earth’s poles (N-North, W-Western, E-Eastern, S-South). According to the task, the animal had to learn the location of an invisible platform (diameter 10 cm) located (between N and E sectors) in one of the cardinal squares of the pool (there are four of them: SW, NW, NE, SE), which was sunk 2 cm from the water surface. The learning (training) procedure lasted 4 days and, consequently, the sequence of starting sectors was shifted with each passing day. In each sample, the animal was randomly placed on one of the four starting sectors (N, S, E, W), facing the wall of the tank. The test time allocated for animals to find the invisible platform was one minute; then, they stayed on the platform for 15 s. If the animal could not find the platform within 1 min, it was placed by the experimenter on the invisible platform for 15 s. In different sessions of the task, the location of the platform was not changed with respect to the orientation in the room environment. The interval between consecutive sections was 2 min, the learning procedure lasted for 4 days, and the daily session consisted of 4 repetitions. Spatial memory was assessed on the fifth day with a test session (between E and S). During this session the hidden platform was removed from the pool. In the test session, the animal was placed in the pool from a new starting point (starting point A) and allowed 1 min to swim. The time spent in each cardinal square of the pool was recorded, which was then used to evaluate memory retention.

The latent period for finding an invisible platform, the time spent in each cardinal square of the pool, and the swimming distance covered by the animal were measured with video surveillance and computer program.

### 4.6. Cell Count

On the 56th day of the experiment, animals were deeply anesthetized with ketamine (100 mg/kg) and then decapitated. The brains were dissected and fixed in 4% paraformaldehyde in 0.1 M phosphate buffer (pH 7.4) for 48 h, then cryoprotected in 30% sucrose solution (approximately for 72 h). For Nissl staining 8–10µm coronal sections of the entire brain were cut on a cryostat (Microm HM 500 M, GMI Ramsey USA). Every 6th section was collected and mounted on a poly-L–lysine coated glass slides. The slides were remained to dry, rehydrated with 100% alcohol, 95% alcohol, and distilled water. Subsequently, the sections were stained in 0.1% Cresyl violet (Sigma-Aldrich, Hamburg, Germany, Cat.N C504) solution. Then slides were washed in distilled water, differentiated in 70% ethyl alcohol, dehydrated in ascending grades of ethyl alcohol and cleared in xylen. Finally, the sections were mounted with DPX and observed under a light microscope (ZEISS, AXIO Lab.A1, Jena, Germany). The number of the cells were estimated in three groups of rats: CON + SAL, KA + SAL and KA + MI. Each group consisted of 6 rats. General morphology and cell count in the hippocampus were assessed by Nissl staining. Cell counting was performed blindly in three hippocampal areas: CA1, CA3 and Dentate Gyrus. For this purpose, the systematic random sampling was used. The 2-dimensional counting grid (250 μm × 250 μm) at the magnification 400x was used and only cells with distinct nucleus and nucleolus were counted. Totally 10–12 sections from each level within experimental and control animals were selected (30 randomly chosen range of visions at the same site of all sections from each animal).

### 4.7. Electrophoresis and Immunoblotting

#### 4.7.1. Subcellular Fractionation

GFAP, LRRC8A subunit of VRAC, PTPRR and doublecortin were determined in the homogenate fraction of hippocampus and neocortex from the following 3 groups of rats: CON + SAL, KA + SAL, KA + MI (5 rats in each group). The rats were decapitated 8 weeks after the starting of experiment, hippocampus and neocortex bilaterally dissected out and frozen immediately on a dry ice. Brain tissue samples were rapidly homogenized in 20 mM Tris-HCl (pH 7.4), 0.32 M sucrose, 1 mM Methylendiamintetraacetic acid, 1 mM sodium orthovanadate, 10 mM sodium pyrophosphate, 0.5 mM ethylene glycol-bis (2-aminoethylether)-N,N,N,N′-tetraacetic acid, and a cocktail of protease inhibitors (Sigma, Hamburg, Germany, Cat.N. P8340).

#### 4.7.2. Protein Determination

The protein concentration was determined in brain tissue homogenate fraction in quadruplicate, using a micro bicinchoninic acid protein assay kit (Pierce, Dallas, TX, USA).

#### 4.7.3. Electrophoresis and Western Immunoblotting

Equal volume aliquots, containing 30 μg protein were applied to the Sodium dodecyl sulphate (SDS) gels and electrophoresis and Western blotting were carried out, as described previously [14]. After the protein had been transferred onto nitrocellulose membranes, the membranes were stained with Ponceau S solution and analyzed with Image J software (https://imagej.net/ImageJ, accessed during the periods of 2019–2021) to confirm uniform gel loading and transfer. Standard immunochemical procedures were carried out using primary antibodies against GFAP (sc-33673; 200 µg/mL; Santa cruz Biotechnology, Heidelber, Germany), LRRC8A (AAC-001; 1:200; Alomone Labs, Jerusalem, Israel), PTPRR (180134; 1:400; Abcam, Cambridge, UK) and Doublecortin (18723; 1 µg/mL, Abcam, Cambridge, UK) and peroxidase-labeled secondary antibodies and Super-Signal West Pico Chemiluminescent substrate (Pierce, Dallas, TX, USA). The optical densities of bands, corresponding to each of this protein were measured using Lab Works 4.0 (UVP). The autoradiographs were calibrated by including in each gel, 4 standards of homogenate (15, 30, 45 and 60 µg of corresponding total protein) obtained from the control rats. Antibodies against Glial fibrillary acidic protein (GFAP), LRRC8A subunit of volume regulated anionic channel (VRAC), protein tyrosine phosphatase receptor type R (PTPRR) and Doublecortin reacted with the protein bands of the following molecular weight 50 KDa, 75 KDa, 75 KDa and 45 KDa correspondingly (Figure 4, Figure 5, Figure 6 and Figure 7). Optical density was proportional to the amounts of GFAP, LRRC8A subunit of VRAC, PTPRR and Doublecortin (see Figure 4B,E, Figure 5B, Figure 6B and Figure 7B). For the data analysis, the optical density of each sample band was divided by optical density of the band for 30 μg of protein standard [16] to give “relative amount of protein”. Supplementary Tables S4–S8 contains original optical density and relative protein amount data.

We have not normalized data with respect to any other housekeeping protein in brain tissue samples, because it cannot be guaranteed that such proteins are not affected by KA treatment [59,60]. We have controlled gel loading by Ponceau S staining, Image J software analysis and calibration with protein standards.

### 4.8. Statistical Methods

#### 4.8.1. EEG Recording

Generalized mixed models were used for analyzing the duration of seizure events, the length of intervals between seizure events, as well as the number of interictal events. Particularly, we used a Poisson linear mixed regression, with group (KA + SAL and KA + MI) as fixed effect and animals as random effect. This model adequately represents the count nature of the data as well as our intention of modelling difference between groups rather than between animals. The Shapiro–Wilk test was used for detecting deviation from normality, while an unpaired, two-tailed *t* test was used for assessing statistical difference in data that did not deviate from normality. All analyses were performed with the R Statistical Software.

#### 4.8.2. Morris Water Maze

Two-way analysis of variance (ANOVA) was used for assessing the statistical significance of factors “day” and “treatment” for the escape latency time, and of factors “quadrant” and “treatment” for the time spent in quadrants I and IV. *p*-values for pairwise comparisons were computed either with the Tukey’s method or with one-tailed *t* tests (corrected with the Benjamini–Hochberg procedure).

#### 4.8.3. Cell Count

Data of cell counts were analyzed by two-way ANOVA with the following factors of experimental condition (CON + SAL, KA + SAL, and KA + MI) and hippocampus subfield (CA1, CA3, and dentate gyrus). Planned comparisons were made between these groups at a defined hippocampus subfield (e.g., CA3). Each group consisted of six animals.

#### 4.8.4. Protein Data

The data were analyzed by one-way ANOVA, with factor–treatment (CON + SAL, KA + SAL and KA + MI). In case of significant effect in ANOVA, planned comparisons were undertaken using a two-tailed *t* test. Each group consisted of five animals from one series of experiments. All statistical tests were two-tailed, and all significant differences are reported.

## 5. Conclusions

MI treatment after KA-induced SE exerts a long-lasting disease-modifying effect as assessed by monitoring the electrographic and behavioral seizures in rats. It also counteracts cell death and preserves morphology of the hippocampus. MI treatment ameliorates epilepsy-related comorbidities, such as deficit in learning and memory, as well as reverts a number of molecular changes associated with epileptogenesis. These effects of MI are still evident even long after the treatment is terminated. The disease-modifying effect of MI could be mediated by presumably a concert of multiple possible mechanisms, including osmolytic, transcriptional and GABergic modules, stabilizing and maintaining normal neuronal circuits and excitability in the hippocampus after epileptogenic insults to the brain.

## Data Availability

The data presented in this study are available on request from the corresponding author R.S.

## Supplementary Materials

The following supporting information can be downloaded at: https://www.mdpi.com/article/10.3390/ijms23031198/s1.

## References

[1] Pitkänen A., Lukasiuk K., Dudek F.E., Staley K.J. (2015). Epileptogenesis. Cold Spring Harb. Perspect. Med..

[2] Fisher R.S., Acevedo C., Arzimanoglou A., Bogacz A., Cross J.H., Elger C.E., Engel J.J., Forsgren L., French J.A., Glynn M. (2014). ILAE official report: A practical clinical definition of epilepsy. Epilepsia.

[3] Becker A.J. (2018). Review: Animal models of acquired epilepsy: Insights into mechanisms of human epileptogenesis. Neuropathol. Appl. Neurobiol..

[4] Loscher W., Brandt C. (2010). Prevention or modification of epileptogenesis after brain insults: Experimental approaches and translational research. Pharmacol. Rev..

[5] Sasa M. (2006). A new frontier in epilepsy: Novel antiepileptogenic drugs. J. Pharmacol. Sci..

[6] Patel D.C., Wilcox K.S., Metcalf C.S. (2017). Novel Targets for Developing Antiseizure and, Potentially, Antiepileptogenic Drugs. Epilepsy Curr..

[7] Solomonia R., Kuchiashvili N., Berulava A., Pkhakadze V., Trapaidze N., Zhvania M., Abesadze I. (2004). Purification and identification of components of the Aquilegia vulgaris extract fraction exhibiting anti-epileptic activity. J. Biol. Phys. Chem..

[8] Sills G.J., Rogawski M.A. (2020). Mechanisms of action of currently used antiseizure drugs. Neuropharmacology.

[9] Fisher S.K., Novak J.E., Agranoff B.W. (2002). Inositol and higher inositol phosphates in neural tissues: Homeostasis, metabolism and functional significance. J. Neurochem..

[10] Solomonia R., Nozadze M., Kuchiashvili N., Bolkvadze T., Kiladze M., Zhvania M., Kigyradze T., Pkhakadze V. (2007). Effect of myo-inositol on convulsions induced by pentylenetetrazole and kainic acid in rats. Bull. Exp. Biol. Med..

[11] Kotaria N., Kiladze M., Zhvania M.G., Japaridze N.J., Bikashvili T., Solomonia R.O., Bolkvadze T. (2013). The protective effect of myo-inositol on hippocamal cell loss and structural alterations in neurons and synapses triggered by kainic acid-induced status epilepticus. Cell. Mol. Neurobiol..

[12] Nozadze M., Mikautadze E., Lepsveridze E., Mikeladze E., Kuchiashvili N., Kiguradze T., Kikvidze M., Solomonia R. (2011). Anticonvulsant activities of myo-inositol and scyllo-inositol on pentylenetetrazol induced seizures. Seizure.

[13] Gamkrelidze G.N., Nanobashvili Z.I., Bilanishvili I.G., Lordkipanidze T., Kandashvili M., Kokaia M., Solomonia R.O. (2019). Concentration- and time-dependent effects of myo-inositol on evoked epileptic afterdischarge in the hippocampus in vivo. Neuroreport.

[14] Solomonia R., Mikautadze E., Nozadze M., Kuchiashvili N., Lepsveridze E., Kiguradze T. (2010). Myo-inositol treatment prevents biochemical changes triggered by kainate-induced status epilepticus. Neurosci. Lett..

[15] Solomonia R., Gogichaishvili N., Nozadze M., Lepsveridze E., Dzneladze D., Kiguradze T. (2013). Myo-inositol treatment and GABA-A receptor subunit changes after kainate-induced status epilepticus. Cell. Mol. Neurobiol..

[16] Tsverava L., Kandashvili M., Margvelani G., Lortkipanidze T., Gamkrelidze G., Lepsveridze E., Kokaia M., Solomonia R. (2019). Long-Term Effects of Myoinositol on Behavioural Seizures and Biochemical Changes Evoked by Kainic Acid Induced Epileptogenesis. Biomed Res. Int..

[17] Frye C.A., Walf A. (2011). Progesterone, administered before kainic acid, prevents decrements in cognitive performance in the Morris Water Maze. Dev. Neurobiol..

[18] Rao M.S., Abd-El-Basset E.M. (2020). dBcAMP Rescues the Neurons From Degeneration in Kainic Acid-Injured Hippocampus, Enhances Neurogenesis, Learning, and Memory. Front. Behav. Neurosci..

[19] Tsverava L., Lordkipanidze T., Lepsveridze E., Nozadze M., Kikvidze M., Solomonia R. (2016). Myoinositol Attenuates the Cell Loss and Biochemical Changes Induced by Kainic Acid Status Epilepticus. Biomed Res. Int..

[20] Brenner M. (2014). Role of GFAP in CNS injuries. Neurosci. Lett..

[21] Seifert G., Carmignoto G., Steinhäuser C. (2010). Astrocyte dysfunction in epilepsy. Brain Res. Rev..

[22] Buccitelli C., Selbach M. (2020). mRNAs, proteins and the emerging principles of gene expression control. Nat. Rev. Genet..

[23] Erkens M., Tanaka-Yamamoto K., Cheron G., Márquez-Ruiz J., Prigogine C., Schepens J.T., Nadif Kasri N., Augustine G.J., Hendriks W.J. (2015). Protein tyrosine phosphatase receptor type R is required for Purkinje cell responsiveness in cerebellar long-term depression. Mol. Brain.

[24] Nateri A.S., Raivich G., Gebhardt C., Da Costa C., Naumann H., Vreugdenhil M., Makwana M., Brandner S., Adams R.H., Jefferys J.G.R. (2007). ERK activation causes epilepsy by stimulating NMDA receptor activity. EMBO J..

[25] Ma T., Wu Y., Chen B., Zhang W., Jin L., Shen C., Wang Y., Liu Y. (2019). D-Serine Contributes to Seizure Development via ERK Signaling. Front. Neurosci..

[26] Curia G., Gualtieri F., Bartolomeo R., Vezzali R., Biagini G. (2013). Resilience to audiogenic seizures is associated with p-ERK1/2 dephosphorylation in the subiculum of Fmr1 knockout mice. Front. Cell. Neurosci..

[27] Jafari R.M., Ghahremani M.H., Rahimi N., Shadboorestan A., Rashidian A., Esmaeili J., Mehr S.E., Dehpour A.R. (2018). The anticonvulsant activity and cerebral protection of chronic lithium chloride via NMDA receptor/nitric oxide and phospho-ERK. Brain Res. Bull..

[28] Jessberger S., Römer B., Babu H., Kempermann G. (2005). Seizures induce proliferation and dispersion of doublecortin-positive hippocampal progenitor cells. Exp. Neurol..

[29] Levesque M., Avoli M. (2013). The kainic acid model of temporal lobe epilepsy. Neurosci. Biobehav. Rev..

[30] Dudek F.E., Staley K.J., Noebels J.L., Avoli M., Rogawski M.A., Olsen R.W., Delgado-Escueta A.V. (2012). The Time Course and Circuit Mechanisms of Acquired Epileptogenesis. Jasper’s Basic Mechanisms of the Epilepsies.

[31] Salami P., Lévesque M., Benini R., Behr C., Gotman J., Avoli M. (2014). Dynamics of interictal spikes and high-frequency oscillations during epileptogenesis in temporal lobe epilepsy. Neurobiol. Dis..

[32] De Curtis M., Avanzini G. (2001). Interictal spikes in focal epileptogenesis. Prog. Neurobiol..

[33] Wang S., Jin B., Yang L., Chen C., Ding Y., Guo Y., Wang Z., Ming W., Tang Y., Wang S. (2017). Clinical value and predictors of subclinical seizures in patients with temporal lobe epilepsy undergoing scalp video-EEG monitoring. J. Clin. Neurosci. Off. J. Neurosurg. Soc. Australas..

[34] McCormick D.A., Contreras D. (2001). On the cellular and network bases of epileptic seizures. Annu. Rev. Physiol..

[35] Bertram E.H., Zhang D., Williamson J.M. (2008). Multiple roles of midline dorsal thalamic nuclei in induction and spread of limbic seizures. Epilepsia.

[36] Moser E.I., Moser M.-B., McNaughton B.L. (2017). Spatial representation in the hippocampal formation: A history. Nat. Neurosci..

[37] Sofroniew M.V. (2014). Astrogliosis. Cold Spring Harb. Perspect. Biol..

[38] Pernot F., Heinrich C., Barbier L., Peinnequin A., Carpentier P., Dhote F., Baille V., Beaup C., Depaulis A., Dorandeu F. (2011). Inflammatory changes during epileptogenesis and spontaneous seizures in a mouse model of mesiotemporal lobe epilepsy. Epilepsia.

[39] Takahashi D.K., Vargas J.R., Wilcox K.S. (2010). Increased coupling and altered glutamate transport currents in astrocytes following kainic-acid-induced status epilepticus. Neurobiol. Dis..

[40] Stringer J.L. (1996). Repeated seizures increase GFAP and vimentin in the hippocampus. Brain Res..

[41] Jentsch T.J. (2016). VRACs and other ion channels and transporters in the regulation of cell volume and beyond. Nat. Rev. Mol. Cell Biol..

[42] Murphy T.R., Binder D.K., Fiacco T.A. (2017). Turning down the volume: Astrocyte volume change in the generation and termination of epileptic seizures. Neurobiol. Dis..

[43] Dietzel I., Heinemann U., Hofmeier G., Lux H.D. (1982). Stimulus-induced changes in extracellular Na+ and Cl- concentration in relation to changes in the size of the extracellular space. Exp. Brain Res..

[44] Rothman S.M. (1985). The neurotoxicity of excitatory amino acids is produced by passive chloride influx. J. Neurosci..

[45] Dudek F.E., Obenaus A., Tasker J.G. (1990). Osmolality-induced changes in extracellular volume alter epileptiform bursts independent of chemical synapses in the rat: Importance of non-synaptic mechanisms in hippocampal epileptogenesis. Neurosci. Lett..

[46] Schwartzkroin P.A., Baraban S.C., Hochman D.W. (1998). Osmolarity, ionic flux, and changes in brain excitability. Epilepsy Res..

[47] Arranz A.M., Perkins K.L., Irie F., Lewis D.P., Hrabe J., Xiao F., Itano N., Kimata K., Hrabetova S., Yamaguchi Y. (2014). Hyaluronan deficiency due to Has3 knock-out causes altered neuronal activity and seizures via reduction in brain extracellular space. J. Neurosci..

[48] Davis S., Laroche S. (2006). Mitogen-activated protein kinase/extracellular regulated kinase signalling and memory stabilization: A review. Genes. Brain. Behav..

[49] Jessberger S., Parent J.M. (2015). Epilepsy and Adult Neurogenesis. Cold Spring Harb. Perspect. Biol..

[50] Pun R.Y.K., Rolle I.J., Lasarge C.L., Hosford B.E., Rosen J.M., Uhl J.D., Schmeltzer S.N., Faulkner C., Bronson S.L., Murphy B.L. (2012). Excessive activation of mTOR in postnatally generated granule cells is sufficient to cause epilepsy. Neuron.

[51] Jakubs K., Nanobashvili A., Bonde S., Ekdahl C.T., Kokaia Z., Kokaia M., Lindvall O. (2006). Environment matters: Synaptic properties of neurons born in the epileptic adult brain develop to reduce excitability. Neuron.

[52] Dai G., Yu H., Kruse M., Traynor-Kaplan A., Hille B. (2016). Osmoregulatory inositol transporter SMIT1 modulates electrical activity by adjusting PI(4,5)P2 levels. Proc. Natl. Acad. Sci. USA.

[53] Neverisky D.L., Abbott G.W. (2017). KCNQ-SMIT complex formation facilitates ion channel-solute transporter cross talk. FASEB J. Off. Publ. Fed. Am. Soc. Exp. Biol..

[54] Bortolatto C.F., Jesse C.R., Wilhelm E.A., Ribeiro L.R., Rambo L.M., Royes L.F.F., Roman S.S., Nogueira C.W. (2011). Protective effect of 2,2′-dithienyl diselenide on kainic acid-induced neurotoxicity in rat hippocampus. Neuroscience.

[55] Racine R.J. (1972). Modification of seizure activity by electrical stimulation. II. Motor seizure. Electroencephalogr. Clin. Neurophysiol..

[56] Clement A.B., Hawkins E.G., Lichtman A.H., Cravatt B.F. (2003). Increased seizure susceptibility and proconvulsant activity of anandamide in mice lacking fatty acid amide hydrolase. J. Neurosci..

[57] Loscher W., Brandt C. (2010). High seizure frequency prior to antiepileptic treatment is a predictor of pharmacoresistant epilepsy in a rat model of temporal lobe epilepsy. Epilepsia.

[58] Paxinos G., Watson C. (1998). The Rat Brain in Stereotaxic Coordinates.

[59] Li R., Shen Y. (2013). An old method facing a new challenge: Re-visiting housekeeping proteins as internal reference control for neuroscience research. Life Sci..

[60] Ghosh R., Gilda J.E., Gomes A. (2014). V The necessity of and strategies for improving confidence in the accuracy of western blots. Expert Rev. Proteom..

