# Supplementary material for: Myo-Inositol Limits Kainic Acid-Induced Epileptogenesis in Rats
Source: Int J Mol Sci. 2022 Jan 21;23(3):1198. doi: 10.3390/ijms23031198 (PMC8835653; doi:10.3390/ijms23031198)
Supplement: Supplementary file 1 [file ijms-23-01198-s001.zip › ijms-1544512 - Supplementary Materials/Supplementary Figure S1 and legend.pdf]

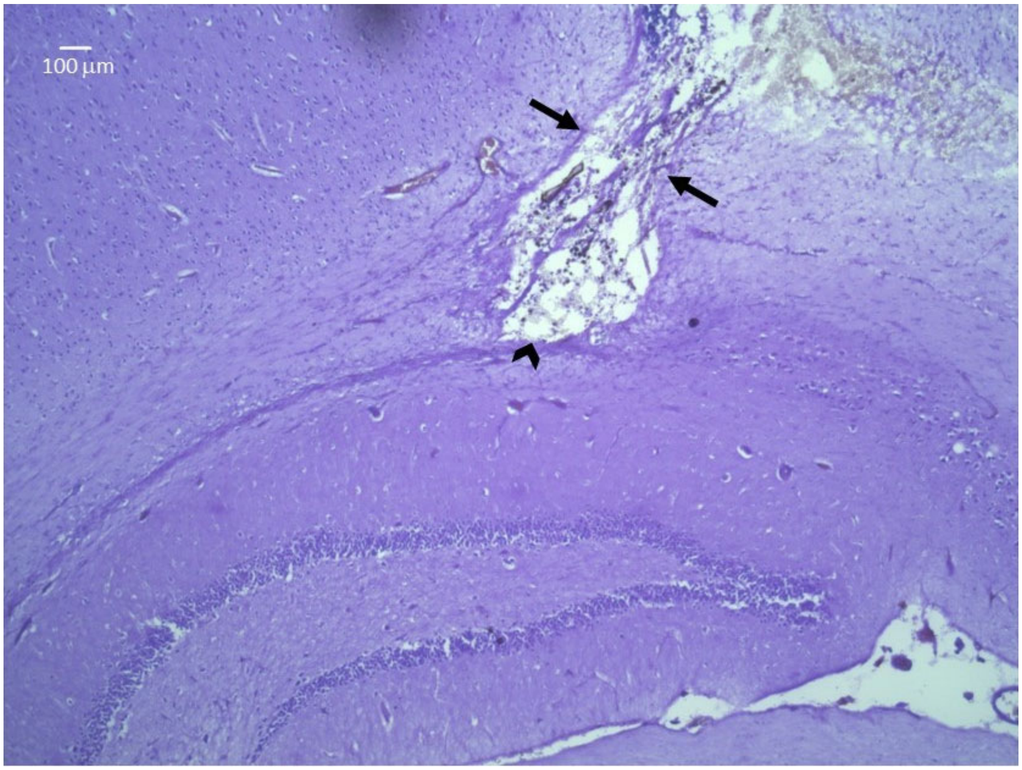

Supplementary Figure S1.

A cross section of the hippocampus indicating field recording electrode location (arrows). The tip of the electrode is located in the molecular layer of the CA1 area (arrowhead).
