# Supplementary material for: Myo-Inositol Limits Kainic Acid-Induced Epileptogenesis in Rats
Source: Int J Mol Sci. 2022 Jan 21;23(3):1198. doi: 10.3390/ijms23031198 (PMC8835653; doi:10.3390/ijms23031198)
Supplement: Supplementary file 1 [file ijms-23-01198-s001.zip › ijms-1544512 - Supplementary Materials/Supplementary Table S1.pdf]

Supplementary Table-S1. Number of spontaneous recurrent seizures (SRS) and their average duration per animal during 8 weeks of observational period.

Animal from KA+SAL group	Number of SRS per animal	Mean duration of SRS per animal	Animal from KA+MI group	Number of seizure electrical activities during the recording period	Mean duration of SRS per animal
KA+SAL-1	20	39.55	KA+MI-1	7	49.28
KA+SAL-1	21	56.71	KA+MI-1	0	0
KA+SAL-1	3	39.33	KA+MI-1	4	33.75
KA+SAL-1	8	57.62	KA+MI-1	0	0
KA+SAL-1	10	40.1	KA+MI-1	3	27.33
KA+SAL-1	10	60.1	KA+MI-1	0	0
KA+SAL-1	7	58.28	KA+MI-1	0	0
Mean ±standard error of the mean	11.29±2.5	50.24±3.8	Mean ±standard error of the mean	2.0±1.0	15.8±7.8
