# Supplementary material for: Myo-Inositol Limits Kainic Acid-Induced Epileptogenesis in Rats
Source: Int J Mol Sci. 2022 Jan 21;23(3):1198. doi: 10.3390/ijms23031198 (PMC8835653; doi:10.3390/ijms23031198)
Supplement: Supplementary file 1 [file ijms-23-01198-s001.zip › ijms-1544512 - Supplementary Materials/Supplementary Table S2.pdf]

Supplementary Table-S2. Number of electrographic seizures per animal during recording period.

Animal from KA+SAL group	Number of seizure electrical activities during the recording period	Animal from KA+MI group	Number of seizure electrical activities during the recording period
KA+SAL-1	291	KA+MI-1	74
KA+SAL-1	83	KA+MI-1	47
KA+SAL-1	149	KA+MI-1	69
KA+SAL-1	167	KA+MI-1	16
KA+SAL-1	176	KA+MI-1	163
KA+SAL-1	189	KA+MI-1	47
KA+SAL-1	100	KA+MI-1	10
Mean ±standard error of the mean	165±26	Mean ±standard error of the mean	60.9±19
