# Supplementary material for: Myo-Inositol Limits Kainic Acid-Induced Epileptogenesis in Rats
Source: Int J Mol Sci. 2022 Jan 21;23(3):1198. doi: 10.3390/ijms23031198 (PMC8835653; doi:10.3390/ijms23031198)
Supplement: Supplementary file 1 [file ijms-23-01198-s001.zip › ijms-1544512 - Supplementary Materials/Supplementary Table S3.pdf]

**Supplementary Table-S3**. Comparison of learning escape latency times within the CON+SAL, KA+SAL and KA+MI groups by days.

| Group of Animals | Comparison between the days | P values | Adjusted P values |
|---|---|---|---|
| Con+SAL | Day 1 vs Day 2 | 0.018 | 0.022 |
| | Day 1 vs Day 3 | 0.0004 | 0.001 |
| | Day 1 vs Day 4 | 8.78E-06 | 0.00005 |
| | Day 2 vs Day 3 | 0.0014 | 0.002 |
| | Day 2 vs Day 4 | 0.0005 | 0.001 |
| | Day 3 vs Day 4 | 0.049 | 0.049 |
| KA+SAL | Day 1 vs Day 2 | 0.005 | 0.015 |
| | Day 1 vs Day 3 | 0.031 | 0.063 |
| | Day 1 vs Day 4 | 0.003 | 0.015 |
| | Day 2 vs Day 3 | 0.97 | 0.97 |
| | Day 2 vs Day 4 | 0.13 | 0.16 |
| | Day 3 vs Day 4 | 0.054 | 0.081 |
| KA+MI | Day 1 vs Day 2 | 0.048 | 0.073 |
| | Day 1 vs Day 3 | 0.011 | 0.035 |
| | Day 1 vs Day 4 | 0.003 | 0.002 |
| | Day 2 vs Day 3 | 0.06 | 0.076 |
| | Day 2 vs Day 4 | 0.02 | 0.04 |
| | Day 3 vs Day 4 | 0.33 | 0.33 |
