# Supplementary material for: Myo-Inositol Limits Kainic Acid-Induced Epileptogenesis in Rats
Source: Int J Mol Sci. 2022 Jan 21;23(3):1198. doi: 10.3390/ijms23031198 (PMC8835653; doi:10.3390/ijms23031198)
Supplement: Supplementary file 1 [file ijms-23-01198-s001.zip › ijms-1544512 - Supplementary Materials/Supplementary Table S6 (A&B).pdf]

Supplementary Table S6A. Hippocampus LRRC8 - optical density data of experimental samples and loaded internal standards.

CON+SAL Group. Animal number	Optical Density	KA+SAL Group. Animal Number	Optical Density	KA+MI Group. Animal Number	Optical Density	Amount of loaded Internal Standard (μg)	Optical Density
CON+SAL-1	241.2	KA+SAL-1	422.1	KA+MI-1	345.980	0	0
CON+SAL-2	242.6	KA+SAL-2	278.8	KA+MI-2	293.022	15	30.4
CON+SAL-3	221.5	KA+SAL-3	471.2	KA+MI-3	247.056	30	169.4
CON+SAL-4	259.9	KA+SAL-4	369.7	KA+MI-4	164.206	45	227.0
CON+SAL-5	376.2	KA+SAL-5	430.8	KA+MI-5	370.000	60	321.2

Supplementary Table S6B. Hippocampus LRRC8 - relative amounts of protein data. These data were calculated from the calibration plot of internal standard protein amount with corresponding optical densities (see Materials and Methods, section 4.7.3. Electrophoresis and Western immunoblotting).

CON+SAL Group. Animal number	Relative amount of protein	KA+SAL Group. Animal Number	Relative amount of protein	KA+MI Group. Animal Number	Relative amount of protein
CON+SAL-1	1.53	KA+SAL-1	2.58	KA+MI-1	2.14
CON+SAL-2	1.54	KA+SAL-2	1.75	KA+MI-2	1.83
CON+SAL-3	1.42	KA+SAL-3	2.86	KA+MI-3	1.56
CON+SAL-4	1.64	KA+SAL-4	2.27	KA+MI-4	1.08
CON+SAL-5	2.31	KA+SAL-5	2.63	KA+MI-5	2.28
