# Supplementary material for: Myo-Inositol Limits Kainic Acid-Induced Epileptogenesis in Rats
Source: Int J Mol Sci. 2022 Jan 21;23(3):1198. doi: 10.3390/ijms23031198 (PMC8835653; doi:10.3390/ijms23031198)
Supplement: Supplementary file 1 [file ijms-23-01198-s001.zip › ijms-1544512 - Supplementary Materials/Supplementary Table S8(A&B).pdf]

Supplementary Table S8A. Hippocampus Doublecortin - optical density data of experimental samples and loaded internal standards.

CON+SAL Group. Animal number	Optical Density	KA+SAL Group. Animal Number	Optical Density	KA+MI Group. Animal Number	Optical Density	Amount of loaded Internal Standard (μg)	Optical Density
CON+SAL-1	225.3	KA+SAL-1	600.0	KA+MI-1	425.8	0	0
CON+SAL-2	236.6	KA+SAL-2	282.7	KA+MI-2	499.4	15	231.1
CON+SAL-3	208.7	KA+SAL-3	448.5	KA+MI-3	461.7	30	538.6
CON+SAL-4	293.2	KA+SAL-4	519.6	KA+MI-4	518.9	45	679.6
CON+SAL-5	288.5	KA+SAL-5	430.3	KA+MI-5	209.0	60	820.6

Supplementary Table S8B. Hippocampus Doublecortin - relative amounts of protein data. These data were calculated from the calibration plot of internal standard protein amount with corresponding optical densities (see Materials and Methods, section 4.7.3. Electrophoresis and Western immunoblotting).

CON+SAL Group. Animal number	Relative amount of protein	KA+SAL Group. Animal Number	Relative amount of protein	KA+MI Group. Animal Number	Relative amount of protein
CON+SAL-1	0.47	KA+SAL-1	1.34	KA+MI-1	0.93
CON+SAL-2	0.49	KA+SAL-2	0.60	KA+MI-2	1.11
CON+SAL-3	0.43	KA+SAL-3	0.99	KA+MI-3	1.02
CON+SAL-4	0.63	KA+SAL-4	1.15	KA+MI-4	1.15
CON+SAL-5	0.61	KA+SAL-5	0.94	KA+MI-5	0.43
